# Effect of mastitis on some hematological and biochemical parameters of Red Sokoto goats

**DOI:** 10.14202/vetworld.2019.572-577

**Published:** 2019-04-19

**Authors:** Bashiru Garba, S. A. Habibullah, Bashir Saidu, Nasiru Suleiman

**Affiliations:** 1 Department of Veterinary Public Health and Preventive Medicine, Faculty of Veterinary Medicine, Usmanu Danfodiyo University, Sokoto, Nigeria; 2 Department of Veterinary Physiology and Biochemistry, Faculty of Veterinary Medicine, Usmanu Danfodiyo University, Sokoto, Nigeria

**Keywords:** biochemical parameters, hematological parameters, mastitis, Red Sokoto goats, Sokoto

## Abstract

**Aim:**

This research project investigates the effect of mastitis on some hematological and biochemical parameters of Red Sokoto goats (RSGs).

**Materials and Methods:**

In this investigation, 16 clinically and subclinically diagnosed mastitic and 20 non-mastitic RSGs, within Sokoto metropolis, were sampled. Blood samples were collected both in ethylenediaminetetraacetic acid and anticoagulant free sample bottles for hematology and biochemical analysis, respectively.

**Results:**

A statistical analysis of the results revealed no significant difference in all the hematological parameters analyzed for both the mastitic and non-mastitic goats except mean corpuscular hemoglobin where significant difference (p<0.05) was observed. Similarly, no significant difference was recorded in the serum biochemistry except for the increase in total protein (p<0.001), globulin (p<0.05), and alanine aminotransferase activity (p<0.05).

**Conclusion:**

This finding is a pointer to the fact that mastitis could be regarded as a localized problem affecting the udder without serious systemic or metabolic involvement in RSGs.

## Introduction

Mastitis is a common disease entity of Red Sokoto goat (RSG), accompanied by physical, chemical, pathological, and bacteriological changes in milk and glandular tissues [1,2]. The disease is usually classified as subclinical, acute, subacute, chronic, and gangrenous based on etiopathological findings and observations [3]. Predisposing factors such as poor management and hygiene, teat injuries, and faulty milking machines are known to hasten the entry of the infectious agents and the course of the disease [4]. Etiologically, the disease is usually incriminated with multifarious agents such as bacteria, mycoplasma, yeast, and other fungi [1,5]. Goat husbandry is becoming increasingly important due to its meat and milk products. Moreover, goat milk provides much-needed animal protein supplement, particularly in developing countries such as Nigeria whose goat milk consumption is gaining importance in some parts of the country [2,6]. These species (goats) constitute about 26.5×10^10^ in Nigeria, the majority of which was located in the extreme northern Sahel region of the country [7,8].

However, management and disease problems, particularly mastitis, constitute major constraints for effective dairy goat husbandry in Nigeria, although the prevalence of other infectious mastitic agents from different locations has been previously reported [2,6]. The cost of mastitis is very apparent for most people working with animals; however, the current costs are not easy to calculate. The total cost of mastitis at herd level can be classified into four fractions, and it is mainly attributed to loss due to depreciation in the quality of milk, loss due to less efficient milk production due to chronic subclinically infected animals, loss due to discharged milk, veterinary fee, and treatment, as well as losses incurred due to increased replacement rate or culling of cows at suboptimal time. However, most important to the farmers and veterinarians are how much these losses can be reduced. The reduction in the loss is one of the hidden benefits in a mastitic control program that will encourage the farmer to improve udder health and make a market for preventive veterinary medicine. Meanwhile, available literature related to the effect of certain infectious diseases on hematological and biochemical parameters in RSGs is rather scarce and only a few authors have looked into the problem. It is an acceptable fact that effective treatment of mastitis depends on accurate diagnosis. The accuracy of diagnosis is largely determined by investigating the hematological and biochemical parameters of animals. Deviation from normal hematobiochemical indices is indicative of ill health, and they aid in diagnosis and evaluation of the severity and prognosis of the disease condition, as evident in the accumulation of white blood cells (WBCs) in mastitic udder [9-11]. In a related development, it has been observed that the WBC is an important and reliable medium in assessing the health status of an animal [12,13]. Similarly, Pradhan *et al*. [14] have reported that physiological and pathological condition of an animal can be assessed through the evaluation of hematological and biochemical parameters of blood. Accordingly, several studies have established normal reference values for hematological and biochemical parameters of indigenous domestic animals in Nigeria.

This study was designed to understand the possible metabolic changes at subclinical and clinical levels of mastitis by assaying serum glucose, cholesterol, total protein, and albumin of RSGs as well as changes in hematological parameters.

## Materials and Methods

### Ethical approval

All animal related procedures including milk and blood sample collection were done in accordance to the recommendations of the Faculty Animal Research Ethics Committee of the Faculty of Veterinary Medicine, Usmanu Danfodiyo University, Sokoto (UDU/FVM/RSG/2018).

### Study area

The samples were collected within Sokoto metropolis, which is located on longitude 11° 3° to 13° east and latitude 4° to 6° north. The vegetation type is Sudan/Sahel Savanna, in which rainfall starts late in May/June to September or early October. The annual rainfall is about 700 mm and relative humidity between 30 and 57%. The Harmattan period is between November and mid-February which is completely without rain but with dust-laden winds that blow from the northwest with a temperature of 11°C in January [15]. The Harmattan is followed by a hot sunny season having temperature ranges between 37 and 43°C with the highest temperature in April. This period results in wide diurnal temperature ranges which varies between 21 and 27°C.

### Sample collection

In this research, 40 RSGs (20 mastitic and 20 apparently healthy) were sampled. All does in various stages of lactation used in this study were managed semi-intensively. Clinical mastitis was defined by the presence of cardinal signs of mastitis and subclinical mastitis was detected after carrying out white side test on the milk samples. The teat orifice was carefully washed and dried with cotton wool; the teat orifice was then scrubbed with 70% alcohol. Approximately 10 ml of milk sample was collected and placed in sterilized glass bottles, cooled immediately, and transferred to the laboratory in an ice box and examined within 24 h of sampling. 1.5 ml and 3.5 ml each of blood samples were collected through the jugular vein in an ethylenediaminetetraacetic acid and anticoagulant free sample bottles for hematological and biochemical analysis, respectively. Sample animals treated with antibiotics by any route within 96 h before the collection were excluded from the study.

### Laboratory examination of milk samples

All the milk samples collected were screened on the spot using Whiteside test as described by Dohoo and Meek [16] with little modification. On a clean glass slide placed over a dark background, 5 drops of milk sample and 2 drops of 4% NaOH were mixed by sterile glass rod for 10 s and then examined for agglutination. Agglutination indicated that the sample was positive for mastitis. However, milk sample that agglutinates in <1 min was graded as 3+, <2 min as 2+, and >2 min as 1+ (indicating severity of the infection). Milk samples that did not agglutinate after the test were designated negative.

### Hematological analyses

Automated QBC” II centrifugal hematology system machine was used for the hematological analysis. The QBC system used a precision sample collection tube that was internally dry coated with reagents such as acridine orange fluorochrome stain and anticoagulants.

Briefly, the hematocrit sample collection tube was filled with 65 µl of blood samples and mixed with the dry reagents in the tube. A precision mechanical expander in the tube expanded the buffy coat permitting digital imaging of the cells. Blue light-emitting diodes illuminated the sample, causing the stained bands of cells within the tube to fluoresce different colors. The fluorescent light then traveled to the camera, where the digital image was captured, and the cell counts, hematocrit, and hemoglobin (Hb) were measured (Drucker Diagnostics™).

The following parameters were evaluated: Packed cell volume, platelet (PLT) count, granulocyte count, WBC count, Hb concentration, mean corpuscular hemoglobin concentration (MCHC), mean corpuscular volume (MCV), and red blood cell (RBC).

### Biochemical assay

The biochemical assay included the following parameters: Glucose, triacylglycerol, cholesterol, high-density lipoprotein (HDL)-cholesterol, total protein, albumin, globulin, alanine aminotransferase (ALT), alkaline phosphatase (ALP), and aspartate aminotransferase (AST) which were all investigated using the Randox commercial assay kits. All the reagents used were of analytical grade. The kits used were procured from commercially available Randox assayed kits Ltd., UK. 3.5 ml of blood taken into anticoagulant free sample bottle was centrifuged at 3000 rpm for 30 min. The supernatant was collected using Pasteur pipette, and the serum was used immediately for the assay. Where the analysis not done immediately, the serum was stored at −20°C until required.

### Statistical analysis

One-way ANOVA with Kramer’s *post hoc* test was performed with the aid of GraphPad InStat version 3.00 (San Diego, California, USA).

## Results

Mean±standard error of the values for the biochemical and hematological parameters are presented in Tables-1 and 2, respectively.

**Table-1:** Some biochemical parameters of mastitic and non-mastitic Red Sokoto goats.

Parameters	Mastitic	Non-mastitic	Mastitic	Non-mastitic	p-value
Glucose (mg/dl)	16 (20)	20 (20)	40.12+7.08	35.63±5.33	NS
TAG (mg/dl)	16 (20)	20 (20)	54.44±5.89	55.75±5.50	NS
Cholesterol (mg/dl)	16 (20)	20 (20)	109.10±9.30	89.17±11.24	NS
HDL cholesterol (mg dl)	16 (20)	20 (20)	49.60±3.43	57.93±4.89	NS
Total protein (g/dl)	16 (20)	20 (20)	8.56±0.25	6.88±0.35	***
Albumin (g/dl)	16 (20)	20 (20)	4.11±0.31	3.46±0.28	NS
Globulin (g/dl)	16 (20)	20 (20)	4.46±0.27	3.41±0.32	*
ALP (U/L)	16 (20)	20 (20)	201.71±36.36	163.67=5.41	*
ALT (U/L)	16 (20)	20 (20)	57.75±1.43	50.50±2.20	*
AST (U/L)	16 (20)	17 (20)	78.18±4.23	91.18±9.54	NS

***p<0.001, *p<0.05, NS=Not significant (p>0.05), ALT=Alanine aminotransferase, TAG=Triacylglycerol, HDL=High-density lipoprotein, ALP=Alkaline phosphatase, AST=Aspartate aminotransferase

**Table-2:** Some hematological parameters of mastitic and non-mastitic Red Sokoto goats.

Parameters	Positive samples	Negative samples	Positive	Negative	p-value
HCT %	12 (20)	20	27.96±0.68	29.59±0.68	NS
Hb (gdf^1^)	11 (20)	20	9.05±0.35	8.78±0.22	NS
RBC×10V	12 (20)	20	4.58±0.20	4.96±0.11	NS
MCHC (gdf^1^)	11 (20)	20	32.18±1.70	29.79±0.13	NS
WBC×10^y^/l	11 (20)	20	12.00±2.18	9.49±1.70	NS
Lymphocyte×10^9^/l	11 (20)	20	3.96±0.55	3.24±0.37	NS
Granulocyte×10^9^/l	11 (20)	20	6.09±2.30	4.04±0.65	NS
MCH pg	11 (20)	20	19.63±1.27	17.32±0.50	*
MCV FI	11 (20)	20	61.58±2.69	59.87±0.12	NS
Platelet×10^y^/l	11 (20)	20	231.64±2.66	233.90±2.04	NS

***p<0.001, *p<0.05, NS=Not significant (p>0.05), Mean difference is significant at p <0.05, *NS=Not significant (p>0.05), Hb=Hemoglobin, MCHC=Mean corpuscular hemoglobin, MCV=Mean corpuscular volume, RBC=Red blood cell

Table-1 shows the mean serum biochemical parameters of mastitic and non-mastitic RSGs. The mean glucose level for mastitic and non-mastitic does was 40.12±7.08/mgdl and 35±5.33/mgdl, respectively. Blood glucose level was higher in mastitic does than non-mastitic does, but the difference was not statistically significant (p>0.05). The mean value recorded for serum cholesterol in mastitic and non-mastitic does was 109.10±9.30/mgdl and 89.17±11.24/mgdl, respectively. Similarly, the mean serum cholesterol level of mastitic does was much higher than the non-mastitic, but the difference was not statistically significant (p>0.05).

The mean HDL cholesterol value for mastic and non-mastitic does was 49.6±3.43/mgdl and 57.93=4.89/mgdl, respectively. The value obtained for mastitic does was lower than that of non-mastitic does, but the difference was not statistically significant (p>0.05).

Serum total protein level in mastitic and non-mastitic does was 8.56±0.25/mgdl and 6.88±0.35/mgdl, respectively. The value obtained for mastitic does was higher than that of the non-mastitic does and the difference is extremely significant (p>0.001). Serum albumin level for mastitic and non-mastitic Red Sokoto does was 4.11±0.31/mgdl and 3.4±0.28/mgdl, respectively. However, there was no significant (p>0.05) difference between the two conditions. The mean globulin level in mastitic and non-mastitic does was 4.46±0.27/mgdl and 3.41±0.32/mgdl, respectively, and the difference between the two conditions is quite significant statistically (p<0.05) with mastitic does having a slightly higher value than the non-mastitic does. The mean value for serum ALP of mastitic and non-mastitic does was 201.71±36.36 U/l and 163.67=5.41 U/I, respectively. The value obtained for mastitic does was higher than that of non-mastitic does, but the difference was not statistically significant (p>0.05).

The mean value of ALT for mastitic and non-mastitic does was 57.75±1.43 U/l and 50.50±2.20 U/l, respectively. The values obtained for mastitic Red Sokoto does were quite higher than that of non-mastitic does, and the difference was quite significant (p<0.05). The mean value of AST in the serum of mastitic and non-mastitic Red Sokoto does was 78.18±4.23 and 91.18±9.54 U/l, respectively. However, the difference is not statistically significant (p>0.05).

As shown in Table-2, the mean values of hematocrit count for mastitic and non-mastitic does were 27.96±1.31% and 29.59±0.68%, respectively. The value obtained for non-mastitic does was slightly higher than that of the mastitic does, but the difference is not statistically significant (p>0.05). The mean values recorded for Hb concentration were 9.05±0.35g/dl for mastitic, and 8.7±0.22 g/dl for non-mastitic does, respectively. Moreover, the value of mastitic does was slightly higher than that of the non-mastitic does, but the difference was not statistically significant (p>0.05). The mean RBC count for mastitic does was slightly lower with 4.58±0.20×10^6^ µl. The difference was also not statistically significant (p>0.05). 32.18±10.70 g/dl and 29.79±0.13 was recorded as mean and standard error values, respectively, of MCHC for both mastitic and non-mastitic does with the mastitic does having slightly higher values though not statistically significant (p<0.05).

The mean WBC counts for mastitic and non-mastitic does were 12.0±2.18×10^9^/I and 9.49±1.70×10^9^/I, respectively. The value obtained for mastitic does was slightly higher than the value recorded for non-mastitic does, but the difference was statistically significant (p>0.05). The mean lymphocyte count for mastitic and non-mastitic does was 3.96±0.55×10^9^/I and 3.2±0.37×10^9^/I, respectively. The value obtained for mastitic does was slightly higher than that of non-mastitic animals, but the difference was not statistically significant (p<0.05).

The granulocyte count for mastitic and non-mastitic does was 6.09×10^9^/l, respectively. The mean value recorded for mastitic does was higher than that of the non-mastitic does, but the difference was not statistically significant (p<0.05) with the mastitic does having a value than the non-mastitic does. The mean value for MCV was 61.58±2.69 fl for mastitic does, while 59.87±0.12 fl was recorded as mean value for non-mastitic does. The value for mastitic does was higher than that of non-mastitic does with no statistically significant difference (p>0.05). The mean PLT counts for mastitic and non-mastitic Red Sokoto does were 231.64±2.66×10^9^/l and 233.90±2.04×10^9^/1, respectively. Mastitic value was slightly lower than the non-mastitic does and the difference was not statistically significant (p>0.05).

## Discussion

Mastitis is a term synonymous with the inflammation of one or multiple of the mammary gland tissues that traditionally precede lactation, especially during the postpartum period [17,18]. Infections are usually characterized by local inflammatory signs, which may be accompanied by systemic signs depending on the severity of the infection. Mastitis is generally considered to be restricted to the affected udder, except in severe cases where systemic illness is observed [19,20].

Nevertheless, recent investigations have revealed that mastitis constitutes a serious problem affecting the reproductive efficiency of ruminants as well as represents a significant public health and economic problem to farmers due to losses incurred and culling [21]. The diagnosis of mastitis in goats is often difficult. This is due to the fact that secretions from affected udder may remain grossly normal and the preliminary investigations based on the somatic cell counts in non-mastitic goats seem higher than the recognized normal range for cows [22]. Nonetheless, routine diagnosis is achieved by physical examination, culture, and susceptibility of the affected tissue and milk sample from the affected gland. In addition, hematological and biochemical analysis, as well as ultrasound, is essential [17,23].

The investigation of basic hematological and biochemical indicators contributes to the knowledge of metabolic profile and their possible disorder, whether of a latent or clinical nature [24,25]. The management system usually predisposes goats to mastitis due to injuries (abrasions, perforations, and ulcerations on teats) [24,26]. The result obtained from the present study showed that mastitis has no significant effect (p>0.05) on hematocrit count, Hb, RBC, MCHC, white blood count, and lymphocyte count. Other parameters not significantly affected (p>0.05) are granulocytes’ MCV and PLT, while mean corpuscular Hb was significantly affected (p<0.05). The mastitic and non-mastitic HCT values of RSG obtained in this stud were 27.96±1.31% and 29.59±0.68%, respectively, which compare favorably with earlier works carried out by Coles who reported values of 26.1±4.1 for healthy goats [27]. The slight difference observed may be due to the season of the year or time of sample collection. There was a significant difference (p<0.05) between mastitic and non-mastitic MCH values with mastitic does having higher MCH values (19.63±1.27) than that of non-mastitic does (17.32±0.50). This is in contrast with the value (21.8±44) reported by Oduye [28] and Egbe-Nwiyi *et al*. [29]. This could probably be due to responsive anemia (hypochromic anemia) in the non-mastitic does indicated by Sastry [30]. Similarly, mastitis appears to have no significant effect (p>0.05) on the total WBC in the RSGs despite the slight increase recorded in the present study. This is not unexpected since it is known that the migration of inflammatory cells is usually associated with acute inflammatory reaction. However, the values recorded in this study are lower than those obtained by Oduye [28]. Although, there was no significant difference (p>0.05) between mastitic and non-mastitic values of lymphocyte, nonetheless, the slight increase noted in mastitic when compared with non-mastitic is not unexpected since an increased inflammatory response is a characteristic of the body’s immune system following invasion by mastitis causing agents. Likewise, there was no statistically significant difference (p>0.05) between mastitic and non-mastitic does in terms of Hb and MCHC values despite the mean of mastitic does being slightly higher in both cases; the values obtained in this study are in consonance with those of Oduye [28] who reported 8.5±1.13 and 33.14±3.4, respectively. This difference is thought to have resulted due to iron deficiency anemia. There was no significant difference (p>0.05) between mastitic and non-mastitic RBC levels although a higher value was recorded in mastitic does. The results obtained in this study are much lower than those obtained by Oduye [28] who recorded the values of 12.3±2.4 for goats. There was also no significant difference between mastitic and non-mastitic does with regard to PLT and MCV values (61.58±2.69 and 59.87±0.12). Interestingly, the MCV values recorded for mastitic and non-mastitic goats in the present study was much higher than the values obtained by Oduye [28] and Coles [27], respectively.

Studies in ewes with mastitis have shown the levels of total protein to be statistically not significant when compared with non-mastitic ewes [31]. In this present study, there was a significant (p<0.001) increase in the level of total protein in favor of the mastitic does. This is thought to have resulted following mobilization of the body’s immune system in response to the inflammatory process taking place in the mammary gland tissue. In this investigation, we also observed the level of serum globulin to be significantly higher (p<0.05) in mastitic goats when compared with non-mastitic does. This may occur due to antibody production in the form of gamma-globulin which is responsible for neutralizing the effect of the invading microorganism [32,33]. There was a statistically significant difference (p<0.05) between mastitic and non-mastitic values of ALT with mastitic does having a higher value than that of non-mastitic does. Increase in serum level of ALT is usually associated with viral hepatitis and myocardial infarction [34]. The result of our study was in contrast with the result obtained for mastitic ewes [35]. Increased level of free fatty acids has been reported to be associated with mastitis as against the findings of this present study; however, the results support earlier findings by El-Deeb [36]. Cholesterol levels in cows with mastitis have been reported to decrease [36]. This is in agreement with the present study, though the increase is not statistically significant (p>0.05) here. Hyperglycemia has been noted in the mastitic does in this study though not statistically significant (p>0.05). This is not surprising since mastitis has been reported to decrease the synthesis of milk lactose and hence the underutilization of serum glucose and the consequent hyperglycemia. This is in contrast with hypoglycemia reported for mastitic ewes by Çetn *et al*. [37] who posited that there is a decrease in food consumption by animals following depression as a result of mastitis. Analysis of serum albumin concentration between mastitic and non-mastitic Red Sokoto does reveals no statistically significant difference (p>0.05). This is despite the fact that values recorded for mastitic does were slightly higher (4.11±0.31) than the non-mastitic does (3.46±0.28). This is in contrast with some previous works that reported a decrease in serum albumin concentration of mastitic ewes [38]. The increase noted in the serum ALP levels of mastitic and non-mastitic was not significant. However, evaluation of ALP level is usually associated with cholesterol levels.

## Conclusion

Although a significant increase in the levels of ALT, total protein, and globulin was observed among the mastitic positive goats, it appears from the present study that mastitis is more of a managemental problem affecting the udder with no serious systemic involvement and hence no much significant metabolic changes in RSGs.

## Authors’ Contributions

BG and SAH conceived and designed the study. BG, BS, and NS participated in sample collection and on spot Whiteside test. BG and SAH carried out the laboratory assays and drafted ad revised the manuscript. All authors read and approved the final manuscript.
